# Newborn Hypoxia/Anoxia Inhibits Cardiomyocyte Proliferation and Decreases Cardiomyocyte Endowment in the Developing Heart: Role of Endothelin-1

**DOI:** 10.1371/journal.pone.0116600

**Published:** 2015-02-18

**Authors:** Alexandra N. Paradis, Maresha S. Gay, Christopher G. Wilson, Lubo Zhang

**Affiliations:** 1 Center for Perinatal Biology, Division of Pharmacology, Department of Basic Sciences, Loma Linda University School of Medicine, Loma Linda, California, United States of America; 2 Division of Neonatology, Loma Linda University School of Medicine, Loma Linda, California, United States of America

## Abstract

In the developing heart, cardiomyocytes undergo terminal differentiation during a critical window around birth. Hypoxia is a major stress to preterm infants, yet its effect on the development and maturation of the heart remains unknown. We tested the hypothesis in a rat model that newborn anoxia accelerates cardiomyocyte terminal differentiation and results in reduced cardiomyocyte endowment in the developing heart *via* an endothelin-1-dependent mechanism. Newborn rats were exposed to anoxia twice daily from postnatal day 1 to 3, and hearts were isolated and studied at postnatal day 4 (P4), 7 (P7), and 14 (P14). Anoxia significantly increased HIF-1α protein expression and pre-proET-1 mRNA abundance in P4 neonatal hearts. Cardiomyocyte proliferation was significantly decreased by anoxia in P4 and P7, resulting in a significant reduction of cardiomyocyte number per heart weight in the P14 neonates. Furthermore, the expression of cyclin D2 was significantly decreased due to anoxia, while p27 expression was increased. Anoxia has no significant effect on cardiomyocyte binucleation or myocyte size. Consistently, prenatal hypoxia significantly decreased cardiomyocyte proliferation but had no effect on binucleation in the fetal heart. Newborn administration of PD156707, an ET_A_-receptor antagonist, significantly increased cardiomyocyte proliferation at P4 and cell size at P7, resulting in an increase in the heart to body weight ratio in P7 neonates. In addition, PD156707 abrogated the anoxia-mediated effects. The results suggest that hypoxia and anoxia *via* activation of endothelin-1 at the critical window of heart development inhibits cardiomyocyte proliferation and decreases myocyte endowment in the developing heart, which may negatively impact cardiac function later in life.

## Introduction

The intrauterine environment plays a well-established role in predisposition to cardiovascular disease later in life [1]. Environmental factors during the critical period of heart development may alter the maturation of the heart and thus potentially its life-long function. Cardiomyocytes are the functional contractile units of the heart that undergo a normal maturation process in which terminal differentiation is the final outcome. As the cardiomyocytes terminally differentiate and exit the cell cycle, they lose their proliferative capacity [2]. Cardiomyocyte growth then transitions from hyperplastic to hypertrophic, in which the cells can only increase in size rather than number [3, 4]. Ultimately the proliferative capacity of cardiomyocytes is lost and the adult heart is known to exhibit negligible increases in cell number [5]. Therefore the timing of this transition is pivotal in determining cardiomyocyte endowment in the heart for the rest of the animal’s life.

Hypoxia is a major stress to preterm infants, yet its effect on the development and maturation of the heart remains unknown. Given that the transition of cardiomyocyte terminal differentiation occurs in rodents during the first two weeks of neonatal life [3, 6], which is an equivalent timeframe to the late fetal stage in third trimester of human gestation [2], they provide a reasonable animal model to study the effect of anoxia on preterm infants at the critical window of the heart development. This process of terminal differentiation begins in the rat heart around postnatal day 4 [3] and progresses until day 14 when the heart is essentially mature, thus three time-points within this period were evaluated in this study. Previous studies in rats have shown that maternal hypoxia (10.5% O_2_) leads to a premature exit from the cell cycle in fetal cardiomyocytes [7–9]. Additionally, neonatal cardiomyocytes have been shown to decrease proliferation when exposed to hypoxic conditions [10]. Studies have also been performed in sheep in which placental restriction is induced, resulting in reduced cardiomyocyte maturation [11] and proliferation [12], increased proportion of mononucleate cardiomyocytes [13], and decreased cardiomyocyte endowment [14]. However, the *in vivo* effects of anoxia, as a preterm model, on cardiomyocyte proliferation and endowment in the developing rat heart are, as of yet, not known. Additionally, the downstream regulators of cardiomyocyte proliferation and maturation are unknown.

Endothelin-1 (ET-1) expression is induced by hypoxia [15–18]. Studies performed in endothelial cells [19, 20] and cardiomyocytes [21] have identified a HIF-1α binding site in the prepro-ET-1 gene. Furthermore, the cardiomyocyte is both a site of synthesis and action for ET-1 [22, 23], as it acts mainly at the paracrine or autocrine level [24, 25]. Our recent work showed that *ex vivo* ET-1 treatment promoted terminal differentiation of fetal cardiomyocytes, *via* an increase in DNA methylation [26]. The predominant ET-1 receptor subtype in cardiomyocytes is the ET_A_-receptor [23], which is thought to be involved in regulating proliferation [24, 27, 28]. Currently, little is known about the role that basal ET-1 plays in the terminal differentiation of cardiomyocytes, as well as the effect of hypoxia/anoxia-induced ET-1 production on this process.

Therefore, in the present study we tested the hypothesis that *in vivo* neonatal anoxia decreases proliferation of cardiomyocytes *via* the ET_A_-receptor-dependent mechanisms, resulting in reduced cardiomyocyte endowment in the developing heart. Herein, we provide evidence that the ET_A_-receptor mediates the anoxia-induced decrease in cardiomyocyte proliferation. Furthermore, cardiomyocyte endowment in the developing heart was decreased by anoxia and restored with PD156707, a selective ET_A_-receptor antagonist.

## Methods

### Experimental animals

Time-dated pregnant Sprague-Dawley rats were purchased from Charles River Laboratories (Portage, MI) and allowed to give birth. Neonatal pups from 7 litters were used and divided into the treatment groups. Data from pups of multiple litters were pooled. Starting at postnatal day 1, newborn rats were placed in a temperature-controlled (37°C) anoxia chamber. Nitrogen was infused into the chamber for 10 minutes and an oxygen sensor was used to verify the level of oxygen in the chamber being < 0.2%. Control animals were placed in a chamber with oxygen maintained at 21%. Anoxia treatments were performed twice a day with 8 hours in between, from postnatal day 1 until postnatal day 3. A group of animals was treated with intraperitoneal injections of an ET_A_-receptor antagonist, PD156707 (2 mg/kg), prior to each episode of anoxia, twice a day for the first 3 postnatal days. Neonatal pups were anesthetized with isoflurane and hearts isolated for studies on postnatal day 4, 7, and 14. To investigate the comparative effect of prenatal hypoxia, some of the time-dated pregnant Sprague-Dawley rats were treated with either normoxic control or 10.5% O_2_ from gestational day 15 to 21, as previously described [29, 30]. Following the hypoxia treatment, pregnant rats were allowed to give birth. Hearts were isolated from postnatal day 4 and 7 neonatal rats. All procedures and protocols were approved by the Loma Linda University Institutional Animal Care and Use Committee (IACUC) and all procedure adhered to the guidelines by US National Institutes of Health Guide for the Care and Use of Laboratory Animals (http://grants.nih.gov/grants/olaw/Guide-for-the-care-and-use-of-laboratory-animals.pdf).

### Measurement of cardiomyocyte number

Hearts from day 4, 7, and 14 neonatal pups were isolated and the atria excised. The hearts were then completely enzymatically digested to yield primary cardiomyocytes, as previously described [26, 31]. A pre-plate step was performed to enrich the cardiomyocyte population. This is a commonly used method [32] that is based on the differential attachment of cardiomyocytes and non-myocyte cells of the heart. Cardiomyocytes take approximately 24 hours to fully attach to the plate while non-myocytes attach within a couple hours. After a 2-hour pre-plate step to remove attached non-myocytes, cardiomyocytes in the media were collected and used for counting cardiomyocyte number *via* a hemacytometer. Briefly, an aliquot of cardiomyocytes was counted using a hemacytometer and the counts were multiplied by the total volume of cell suspension and normalized according to the heart weight, to yield the number of cardiomyocytes per heart weight.

### Immunocytochemistry

To perform immunocytochemical staining, cardiomyocytes isolated from day 4 and 7 hearts were allowed to attach to plates in Hyclone Medium 199 (Thermo Scientific) supplemented with 10% fetal bovine serum (Gemini Bio-Products) and 1% antibiotics (10,000 I.U./mL penicillin, 10,000 μg/mL streptomycin) at 37°C in 95% air/5% CO_2_. After 24 hours, cardiomyocytes were fully attached and were double stained with alpha-actinin, a cardiomyocyte marker, and Ki-67, a proliferation marker as described previously [8, 26]. Cardiomyocytes were plated on coverslips and fixed with 4% paraformaldehyde (ThermoScientific) for 15 minutes followed by permeabilization with Triton X-100 (Fisher) for 10 minutes. The cells were blocked with 1% bovine serum albumin for 1 hour at room temperature before incubation with the primary antibodies: mouse anti-α-sarcomeric actinin (A7811, Sigma) (1:200) and rabbit anti-Ki-67 (ab16667, Abcam) (1:100) at room temperature for 1 hour. The samples were incubated with the secondary antibodies: anti-mouse Alexa Fluor 488 (A21202, Life Technologies) and anti-rabbit Alexa Fluor 647 antibodies (A21244, Life Technologies) for 1 hour at room temperature. Nuclei were stained with Hoescht (Sigma) for less than 1 minute. The immunofluorescence staining was assessed using a Zeiss Axio Imager.A1 microscope and quantitative analysis was carried out using *ImageJ* software (http://imagej.nih.gov/ij/). Ki-67 expression, binucleation, and cell size were measured.

### Flow cytometry

Primary cardiomyocytes isolated from day 14 neonatal rats were stained for analysis by flow cytometry. Cells were washed in staining buffer (PBS + 5% FBS), spun down, and re-suspended in 4% paraformaldehyde for 20 minutes at room temperature in the dark. The fixed cells were then washed in permeabilization wash buffer (eBioscience) and supernatant discarded. Cells were stained with antibodies for the cardiomyocyte marker, Troponin T (ab10214, Abcam) (1:200), and proliferation marker, Ki-67-conjugated to allophycocyanin (APC) (eBioscience) (50–5698, 1:200). After incubation and washing, cells were incubated with the secondary antibody for Troponin T, fluorescein isothiocyanate (FITC) (555988, BD Pharmingen) (1:100). Finally cells were washed and resuspended in 1% paraformaldehyde to be run on a FACSAria (BD Biosciences) and analyzed via FACSDiva software (BD Biosciences) for percentage of Ki-67 expressing cardiomyocytes.

### Quantitative real-time PCR

RNA was isolated from the postnatal day 4 (P4) hearts and prepro-ET-1 mRNA abundance was determined by real-time RT-PCR using Icycler Thermal cycler (Bio-Rad), as described previously [30]. Reverse transcription and cDNA synthesis was performed using SuperScript III First-Strand Synthesis Supermix for RT-PCR (Life Technologies). The primers are 5’-CTAGGTCTAAGCGATCCTTGAA-3’ (forward) and 5’-CTTGATGCTGTTGCTGATGG-3’ (reverse). PCR was performed in triplicate, and threshold cycle numbers were averaged.

### Western immunoblotting

HIF-1α, ET_A_-receptor (ET_A_R), and ET_B_-receptor (ET_B_R) protein abundance in the P4 heart was measured from control and anoxia groups. The protein abundance of cyclin D2 and p27 was measured in P4 hearts from control and anoxia groups as well as in the presence and absence of PD156707. Tissues were homogenized and protein isolated using the RIPA lysis buffer system (Santa Cruz Biotechnology). Protein concentrations were quantified using the BCA protein assay (ThermoScientific) and all samples were loaded with equal protein onto 7.5% (HIF-1α) or 10% (ET_A_R, and ET_B_R, cyclin D2, and p27) polyacrylamide gel with 0.1% sodium dodecyl sulfate (SDS). Proteins were then separated by electrophoresis and transferred onto nitrocellulose membranes. Non-specific binding sites were blocked with Tris-buffered saline solution (TBS) containing 5% dry milk. The membranes were incubated with primary antibodies against HIF-1α (sc10790, Santa Cruz Biotechnology; 1:500), ET_A_R (sc33536, Santa Cruz Biotechnology; 1:500), ET_B_R (sc33538, Santa Cruz Biotechnology; 1:500), cyclin D2 (ab3085, Abcam; 1:1000), and p27 (ab7961, Abcam; 1:1000). After washing, membranes were incubated with secondary antibodies. Proteins were visualized with enhanced chemiluminescence reagents and western blots were exposed to Hyper film. Kodak image software was used to quantify all results.

### Statistical analysis

Data are expressed as means ± SEM. Statistical analysis (p < 0.05) was determined by two-way analysis of variance (ANOVA) followed by Neuman-Keuls *post hoc* test or Student’s t test, where appropriate, using GraphPad Prism software. The two-way ANOVA was performed to evaluate the effects of two factors, within each age group: (1) control versus anoxia, and (2) in the presence and absence of PD156707.

## Results

### Newborn anoxia treatment increased pre-proET-1 mRNA in the heart of P4 neonate

**Fig 1:**
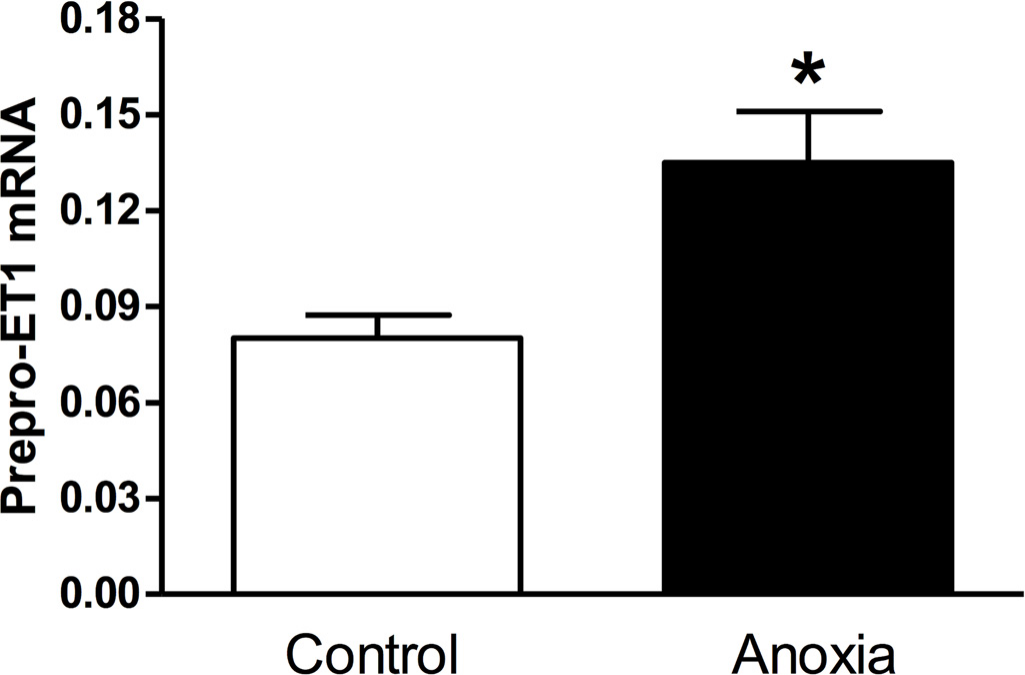
Effect of newborn anoxia on prepro-ET-1 mRNA in the neonatal heart. Hearts were isolated from day 4 neonatal rats treated with control or anoxia. mRNA abundance of prepro-ET-1 was determined by real-time RT-PCR. Data are means ± SEM. * P < 0.05, anoxia *vs*. control. n = 3–5.

Neonatal rats were exposed to anoxia twice a day from postnatal day 1 to 3, and hearts were isolated at P4. As seen in Fig. 1, there was a significant increase in prepro-ET-1 mRNA abundance in neonatal hearts exposed to anoxia (< 0.2% O_2_), as compared to the normoxic control (21% O_2_).

### Newborn anoxia treatment increased HIF-1α protein abundance in the heart of the P4 neonate

**Fig 2:**
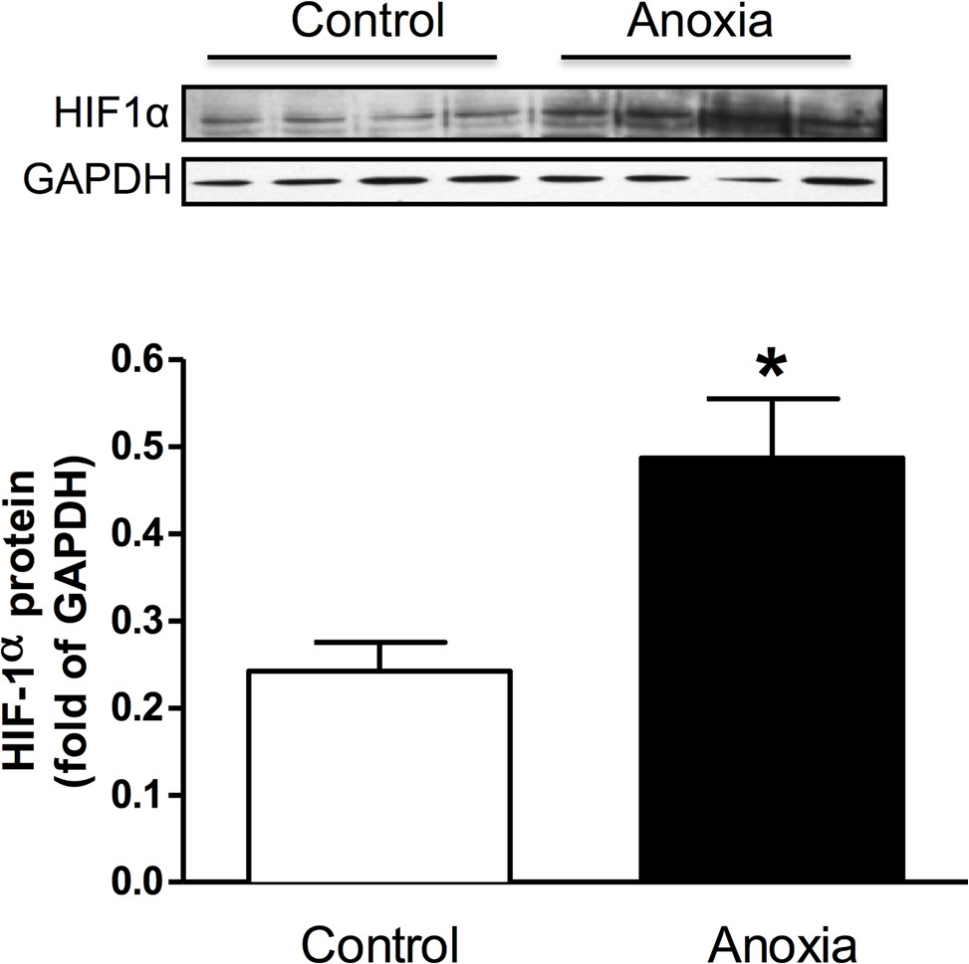
Effect of newborn anoxia on HIF-1α protein abundance in the neonatal heart. Hearts were isolated from day 4 neonatal rats treated with control or anoxia. Protein abundance of HIF-1**α** was determined by Western immunoblotting. Data are means ± SEM. * P < 0.05, anoxia *vs*. control. n = 4.

Hearts from P4 rats treated with anoxia were collected and protein isolated. Neonatal hearts exposed to anoxia had significantly increased levels of the HIF-1α protein (Fig. 2).

### Newborn anoxia treatment decreased cardiomyocyte proliferation

**Fig 3:**
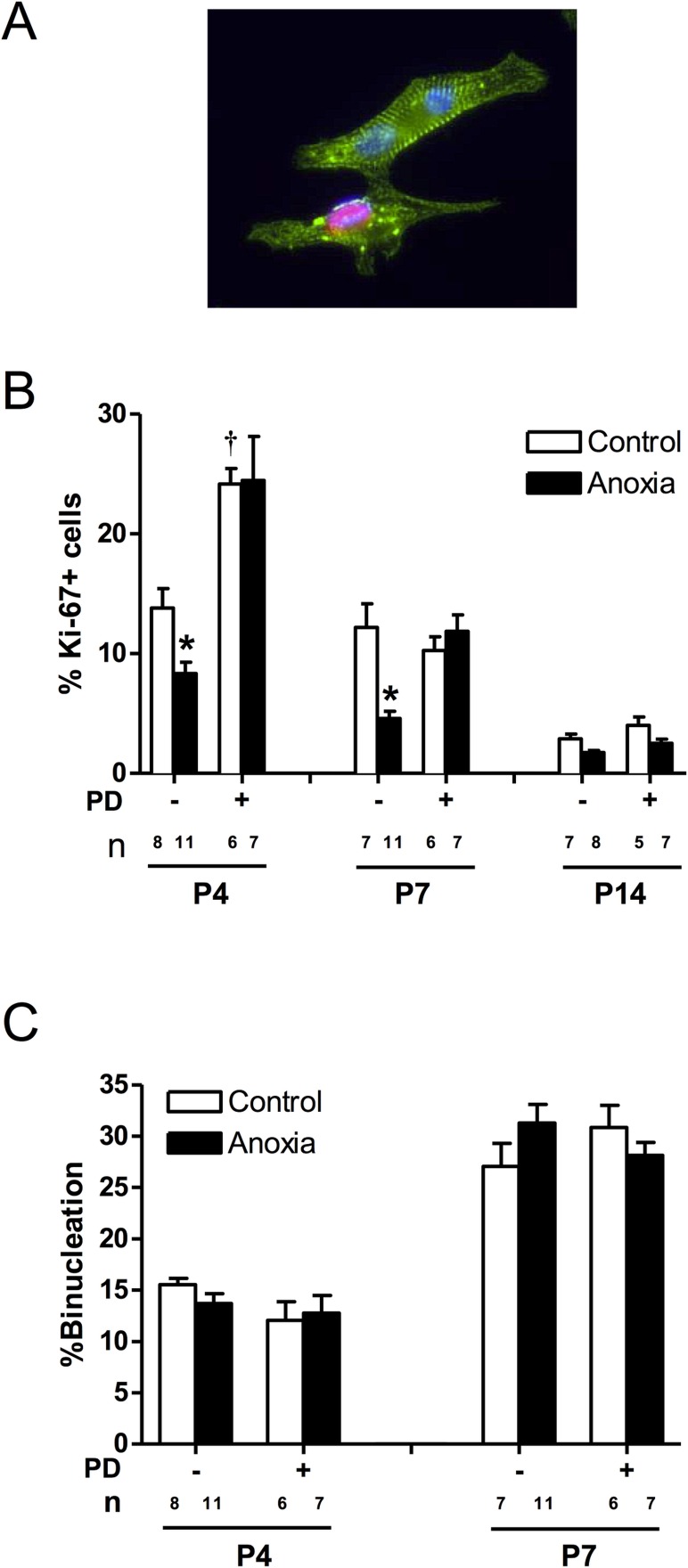
Effect of newborn anoxia and PD156707 on proliferation and binucleation of neonatal cardiomyocytes. Cardiomyocytes were isolated from P4, P7, and P14 neonatal rats that were treated with control or anoxia, in the absence or presence of PD156707. Cells from P4 and P7 rats were stained with α-actinin and Ki-67, and nuclei were stained using Hoechst staining. P14 cardiomyocytes were stained with Ki-67 and analyzed *via* FACS. **Panel A** shows a representative image of cardiomyocytes stained with **α**-actinin (green), Ki-67 (red), and Hoescht (blue). **Panel B** shows percent of Ki-67 expressing cells. **Panel C** shows percent of binucleate cells. Data are means ± SEM. * P < 0.05, anoxia *vs*. control. † P < 0.05, -PD156707 *vs*. +PD156707. PD: PD156707; n: animal numbers.

As shown in Fig. 3, there is a development-dependent decrease in cardiomyocyte proliferation at the critical window of the heart development during the first two weeks of life in rodents, and myocyte proliferation reduces to minimal levels at postnatal day 14. Anoxia treatment of newborns caused a significant decrease in the proliferation of neonatal cardiomyocytes at both postnatal days 4 and 7 (Fig. 3B). Treatment of newborn rats with a selective ET_A_-receptor antagonist, PD156707, caused a significant increase in cardiomyocyte proliferation in P4 neonatal rats (Fig. 3B). In addition, PD156707 abrogated the anoxia-induced effects in the developing hearts (Fig. 3B). In contrast to proliferation, there is a development-dependent increase in cardiomyocyte binucleation in the developing heart (Fig. 3C). Neither anoxia nor PD156707 treatments had significant effects on cardiomyocyte binucleation (Fig. 3C).

### Newborn anoxia treatment decreased cyclin D2 and increased p27 expression in the heart of the P4 neonate

**Fig 4:**
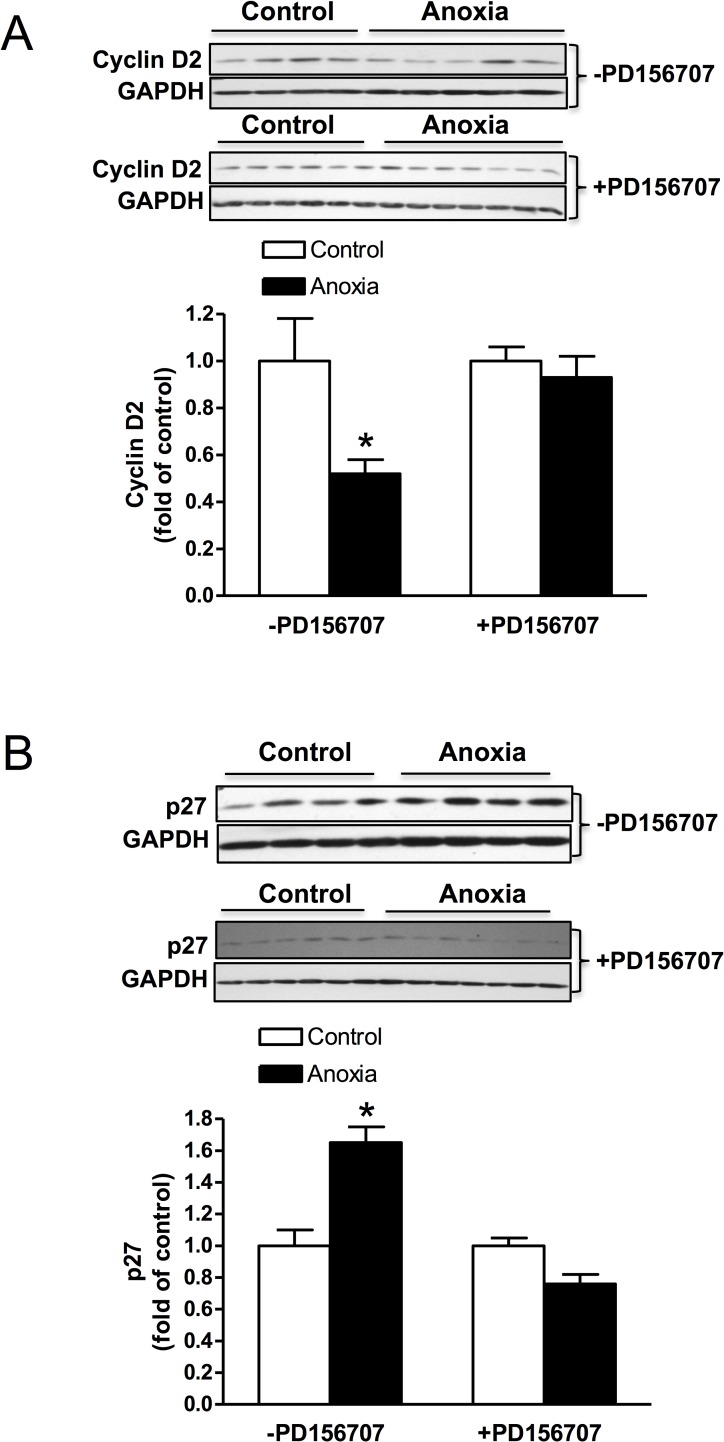
Effect of newborn anoxia on cyclin D2 and p27 protein expression in the cardiomyocyte. Hearts were isolated from day 4 neonatal rats treated with control or anoxia in the presence (n = 6–7) or absence (n = 4) of PD156707. Protein abundance of cyclin D2 in the absence and presence of PD156707 (A), and p27 in the absence and presence of PD156707 (B) was determined by Western immunoblotting. Data are means ± SEM. * P < 0.05, anoxia *vs*. control.

The protein expression of cyclin D2 was decreased due to anoxia treatment and this effect was abolished in the presence of PD156707 (Fig. 4A). On the contrary, p27 expression in the neonatal heart was significantly increased due to anoxia treatment, and PD156707 blocked the effect of anoxia (Fig. 4B).

### Newborn anoxia treatment decreased cardiomyocyte number by day 14

**Fig 5:**
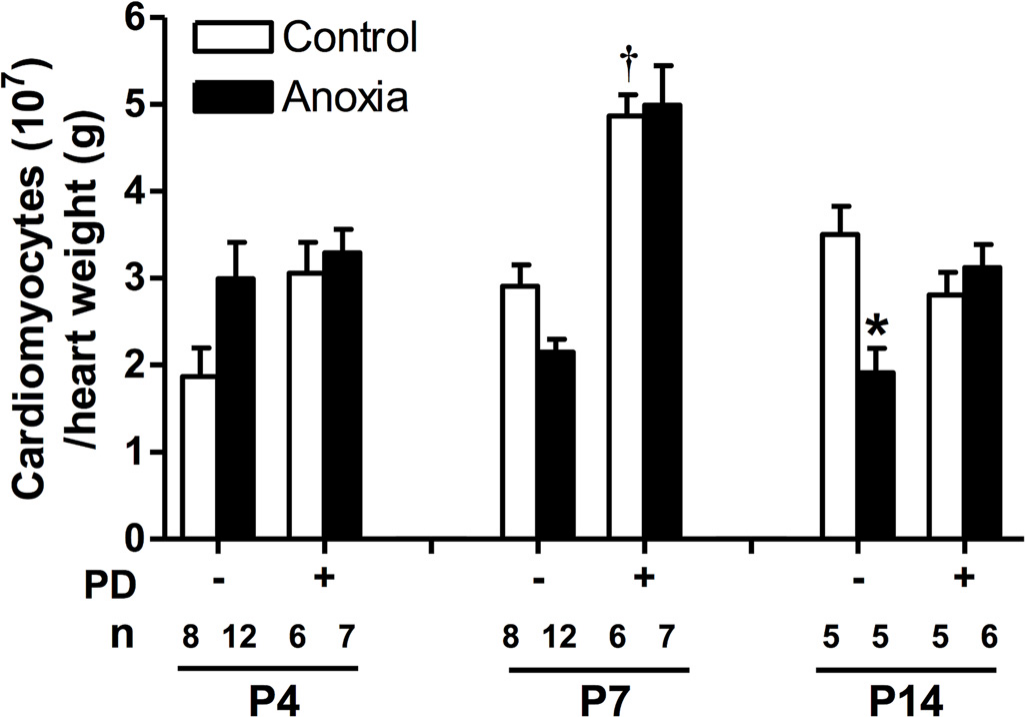
Effect of newborn anoxia and PD156707 on number of cardiomyocytes per heart weight. Cardiomyocytes were isolated from day 4, 7, and 14 neonatal rats that were treated with control or anoxia, in the absence or presence of PD156707. Hearts were weighed and cardiomyocytes counted by hemacytometer. Data are expressed as cardiomyocyte number/g heart weight, and are means ± SEM. * P < 0.05, anoxia *vs*. control. † P < 0.05, -PD156707 *vs*. +PD156707. PD: PD156707; n: animal numbers.

There was no significant change in cardiomyocyte number due to newborn anoxia treatment at day 4 and 7. However, results for day 14 show that anoxia leads to a significant decrease in cardiomyocyte number per heart weight (Fig. 5). PD156707 alone caused a significant increase in cardiomyocyte number in the day 7 neonate (Fig. 5). In the presence of PD156707, the anoxia-mediated effects at day 14 were blocked (Fig. 5).

### Cell size was increased in the presence of PD156707

**Fig 6:**
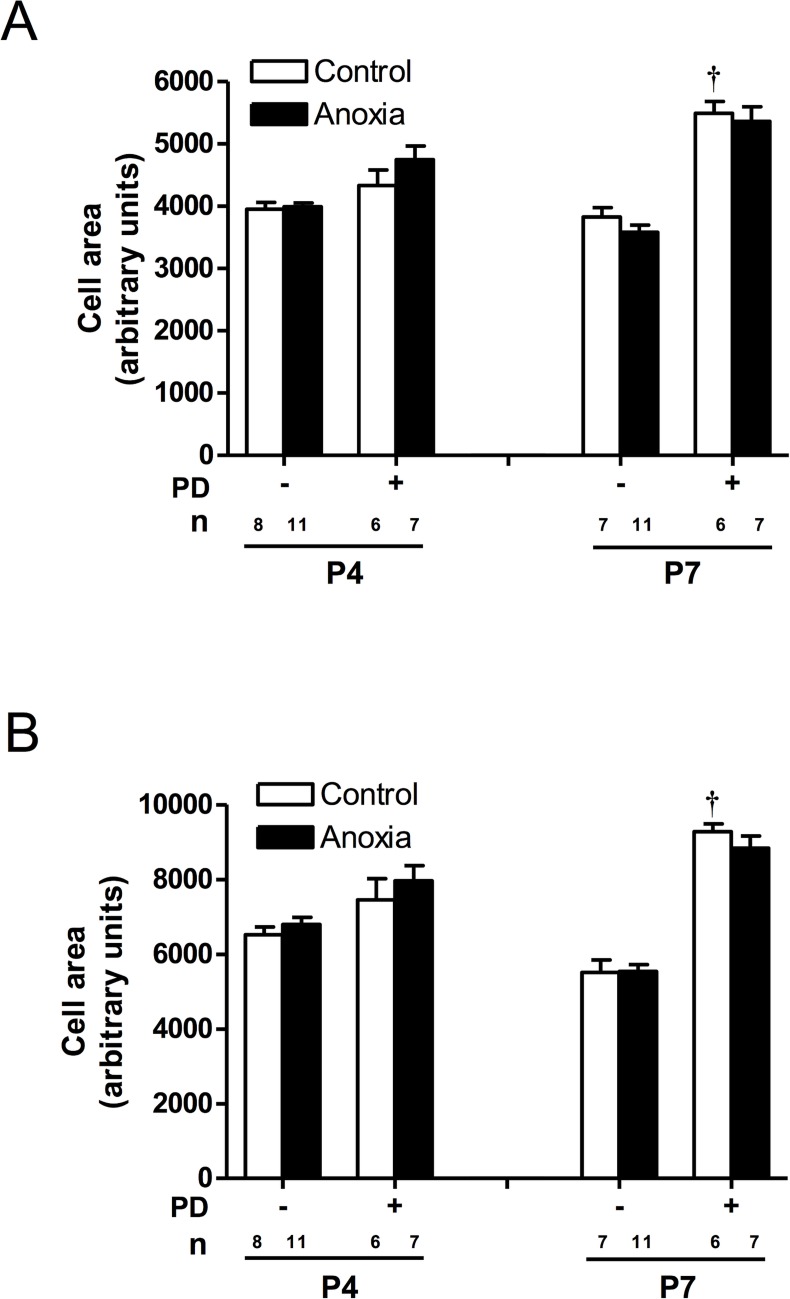
Effect of newborn anoxia and PD156707 on cardiomyocyte size in the neonatal heart. Cardiomyocytes were isolated from day 4, 7, and 14 neonatal rats that were treated with control or anoxia, in the absence or presence of PD156707. Mononucleate (A) and binucleate (B) cell size was measured using *ImageJ*. Data are means ± SEM. † P < 0.05, -PD156707 *vs*. +PD156707. PD: PD156707; n: animal numbers.

Anoxia had no effect on mononucleate or binucleate cell size at either day 4 or 7 (Fig. 6). However, PD156707 treatment was able to increase both mononucleate and binucleate cell size at postnatal day 7 (Fig. 6).

### PD156707 increased heart to body weight ratio

**Fig 7:**
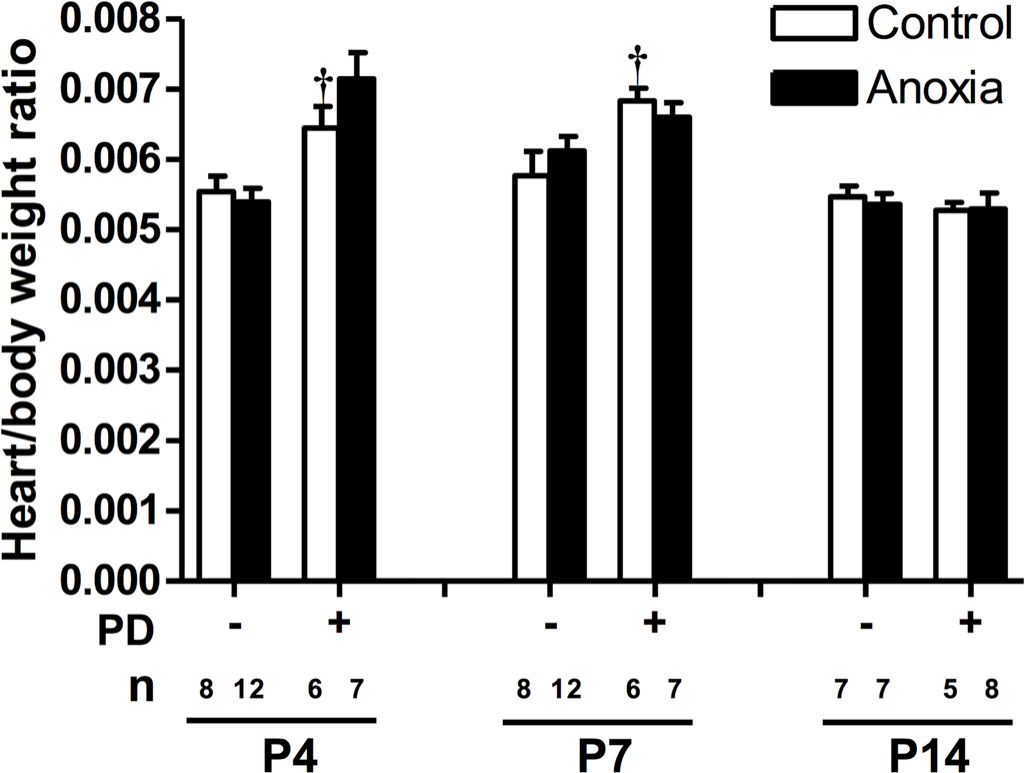
Effect of newborn anoxia and PD156707 on heart to body weight ratio of neonatal rats. Body and heart weights were taken from day 4, 7, and 14 neonatal rats that were treated with control or anoxia, in the absence or presence of PD156707. Data are means ± SEM. † P < 0.05, -PD156707 *vs*. +PD156707. PD: PD156707; n: animal numbers.

There was no significant effect of anoxia on the heart to body weight ratio for any day (Fig. 7). However, PD156707 treatment significantly increased the heart to body weight ratio in day 4 and 7 neonates (Fig. 7). Heart and body weight averages in the presence and absence of anoxia and PD156707 are listed in Table 1.

**Table 1:** Effect of newborn anoxia and PD156707 on body and heart weight of neonatal rats.

		Body weight (grams)			Heart weight (grams)			Heart to Body weight ratio		
		P4	P7	P14	P4	P7	P14	P4	P7	P14
-PD156707	Control	8.51 ± 0.374 (8)	14.44 ± 0.550 (8)	22.45 ± 1.090 (7)	0.047 ± 0.0023 (8)	0.083 ± 0.0046 (8)	0.123 ± 0.0076 (7)	0.0055 ± 0.0002 (8)	0.0058 ± 0.0003 (8)	0.0055 ± 0.0002 (7)
	Anoxia	8.06 ± 0.317 (12)	13.65 ± 0.472 (12)	22.28 ± 0.879 (7)	0.044 ± 0.0023 (12)	0.083 ± 0.0025 (12)	0.120 ± 0.0072 (7)	0.0054 ± 0.0002 (12)	0.0061 ± 0.0002 (12)	0.0054 ± 0.0002 (7)
+PD156707	Control	9.57 ± 0.186 (6)	15.05 ± 0.289 (6)	21.68 ± 0.960 (5)	0.062 ± 0.0029 (6)	0.103 ± 0.0024* (6)	0.114 ± 0.0053 (5)	0.0065 ± 0.0003 (6)	0.0068 ± 0.0002* (6)	0.0053 ± 0.0001 (5)
	Anoxia	7.57 ± 0.498 (7)	12.75 ± 0.302 (7)	23.90 ± 0.779 (8)	0.054 ± 0.0042 (7)	0.084 ± 0.0034 (7)	0.127 ± 0.0081 (8)	0.0072 ± 0.0004 (7)	0.0066 ± 0.0002 (7)	0.0053 ± 0.0002 (8)

Body and isolated hearts were weighed from day 4, 7, and 14 neonatal rats that were treated with control or anoxia, in the absence or presence of PD156707. Heart to body weight ratio values are also represented. Data are means ± SEM.

* P < 0.05, control-PD156707 *vs*. +PD156707.

Number of animals is represented in parentheses.

### Neonatal anoxia treatment had no effect on ET-receptor density

**Fig 8:**
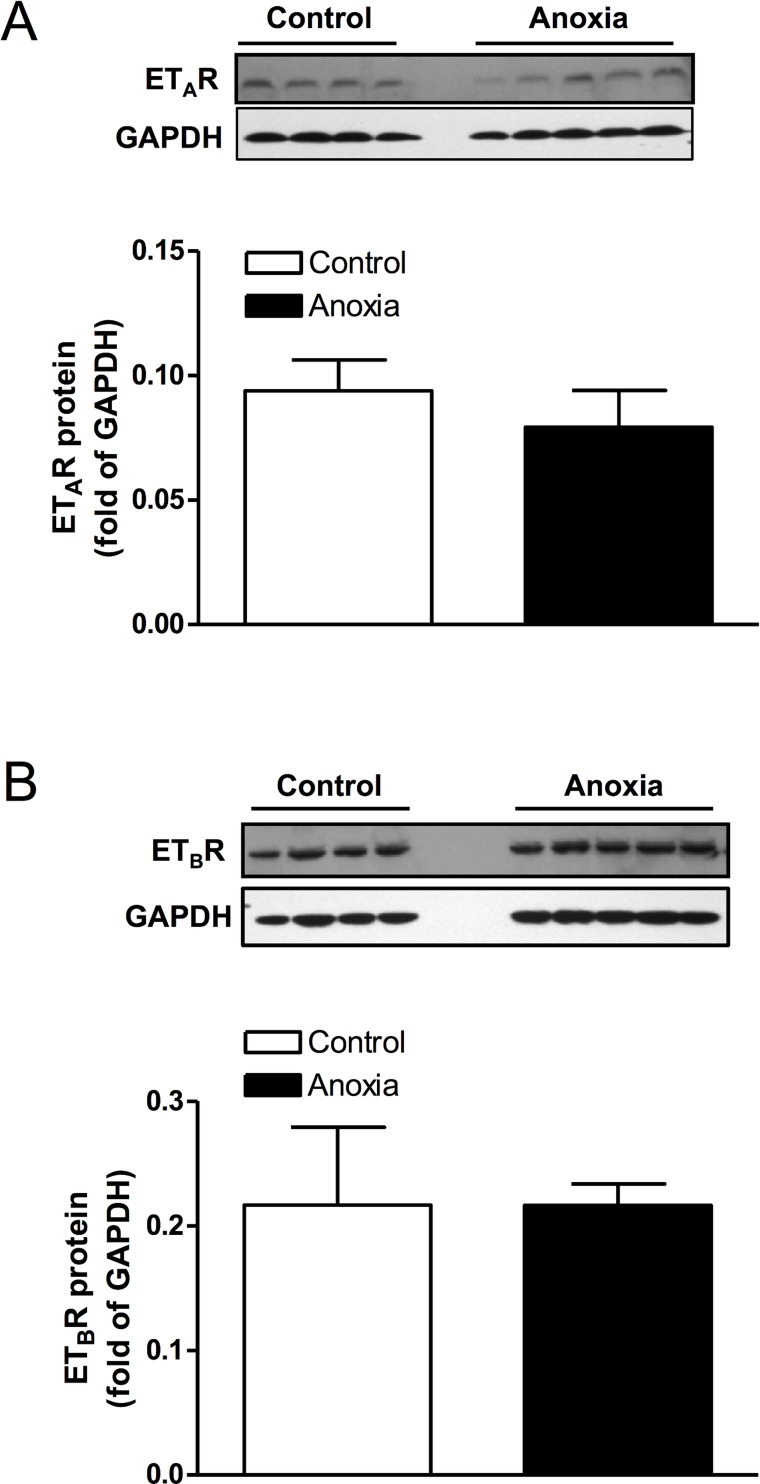
Effect of newborn anoxia on ET_A_- and ET_B_-receptor protein abundance in the neonatal heart. Hearts were isolated from day 4 neonatal rats treated with control or anoxia. Protein abundance of ET_A_R (A) and ET_B_R (B) was determined by Western immunoblotting. Data are means ± SEM. n = 4–5.

Hearts from postnatal day 4 rats that were treated with anoxia were collected and protein isolated. There was no significant change in protein abundance of either ET_A_R or ET_B_R, due to anoxia treatment (Fig. 8).

### Prenatal hypoxia decreased cardiomyocyte proliferation in the fetal heart

**Fig 9:**
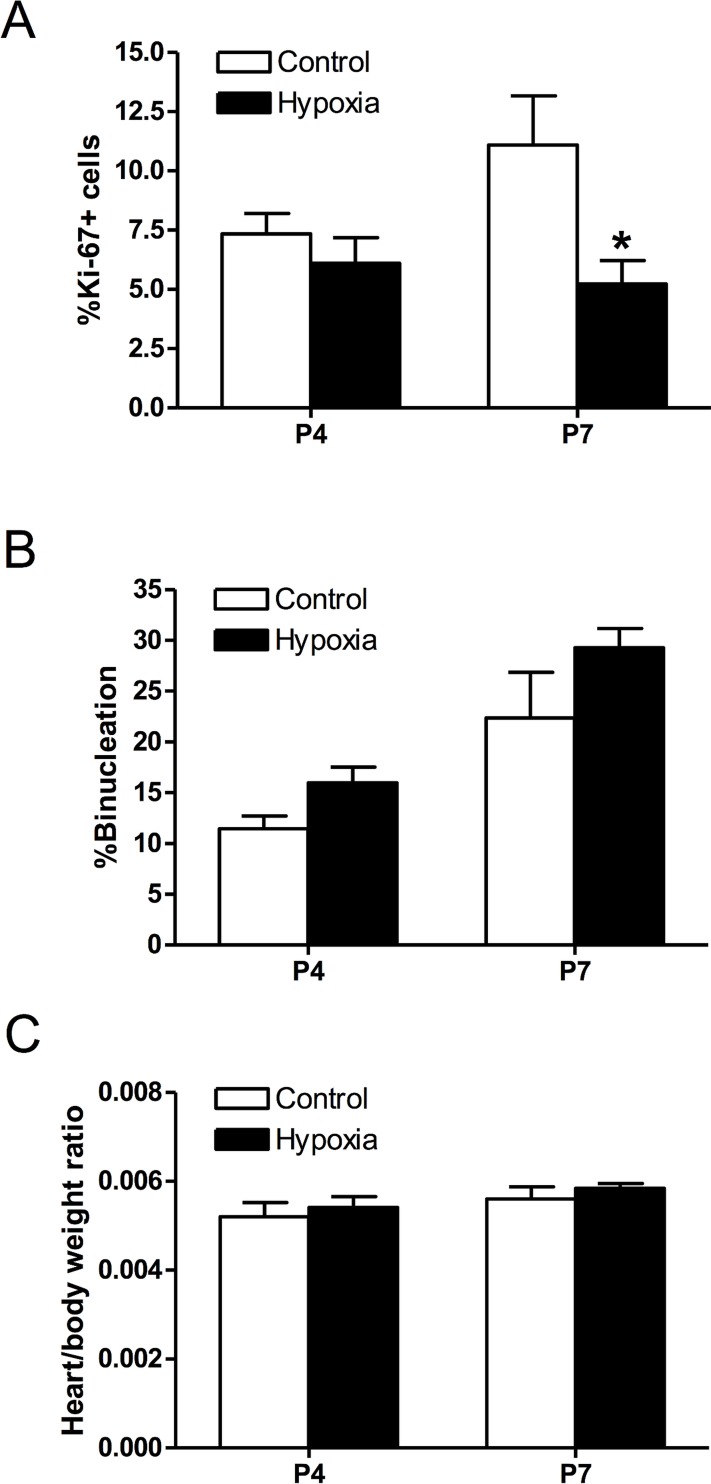
Effect of prenatal hypoxia on neonatal cardiomyocyte proliferation, binucleation, and heart to body weight ratio. Cardiomyocytes were isolated from day 4 and 7 neonatal rats that were treated with control or maternal hypoxia. Cells were stained with α-actinin and Ki-67, and nuclei stained with Hoechst. **Panel A** shows percent of Ki-67 expressing cells (n = 3–4). **Panel B** shows percent of binucleate cells (n = 4). **Panel C** shows the heart to body weight ratio (n = 8–9). Data are means ± SEM. * P < 0.05, hypoxia *vs*. control.

To investigate the comparative effect of prenatal hypoxia, pregnant rats were treated with either normoxic control or 10.5% O_2_ from gestational day 15 to 21, and hearts were isolated from postnatal day 4 and 7 neonatal rats. Similar to the findings in newborn anoxia treatment, prenatal hypoxia resulted in a significant decrease in the proliferation of cardiomyocytes at postnatal day 7 (Fig. 9A), but had no significant effects on percent binucleation (Fig. 9B) or the heart to body weight ratio (Fig. 9C).

## Discussion

In the present study, we provide evidence showing that *in vivo* newborn anoxia leads to a decrease in the proliferation of cardiomyocytes in the developing heart. Furthermore, our results suggest that anoxia treatment leads to a significant reduction in number of cardiomyocytes per heart weight of the day 14 neonate, which is terminally differentiated. The findings that anoxia increased ET-1 production in the heart and the anoxia-induced changes in proliferation and cardiomyocyte number were reversed with PD156707, suggest a mechanism mediated by the ET_A_-receptor. In addition, basal ET-1 was also found to play a role in cardiomyocyte proliferation, as well as the heart to body weight ratio.

Cardiomyocytes undergo a terminal differentiation process that reaches completion by the first two weeks of neonatal life in rats [2, 6]. After this, cardiomyocytes in the heart have negligible proliferative capacity and further growth is mainly *via* hypertrophy. Thus the number of cardiomyocytes that will reside in the adult heart is determined during this early stage and if altered may result in life-long consequences. Hypoxic stress during perinatal development has been shown by previous studies to diminish the proliferation of cardiomyocytes [8, 9, 26]. Furthermore, fetal hearts exposed to hypoxia have fewer [7, 14] and larger cardiomyocytes [7], and adult male rats that were exposed to hypoxia *in utero* were more susceptible to ischemic injury as seen by increased myocardial infarction and reduced recovery [33].

Preterm birth is a complex clinical problem that is highly associated with episodes of severe hypoxia and even anoxia, which can be so severe that the infant must be mechanically ventilated [34]. Preterm infants have an immature respiratory system [35] that is unable to provide adequate oxygen at times and thus ventilatory support is frequently needed. However, several studies have shown that episodic airway obstruction and hypoxemia commonly occur in these infants [36, 37]. Given that the rodent heart is relatively immature at birth, the present study with episodic anoxia treatments of newborn rats provides a reasonable animal model to study the effects of anoxia/hypoxia on the heart development in preterm infants. Anoxia itself has been shown to alter proliferation, and, in rat fibroblasts, leads to arrest of the cell cycle at the G1 and S phase [38].

To confirm the extent of hypoxic exposure to the neonatal hearts used in our study, the protein levels of hypoxia-inducible factor 1 alpha (HIF-1α) were evaluated. The results show that HIF-1α protein abundance is significantly increased in neonatal hearts exposed to *in vivo* anoxia; furthermore increased levels of HIF-1α in the heart have also been observed in the prenatal hypoxia model [7]. In agreement with previous work, we showed that cardiomyocyte proliferation was decreased following *in vivo* neonatal anoxia treatment at postnatal day 4 and 7. The cardiomyocytes take approximately 24 hours for them to fully attach to the plate before the immunocytochemical staining of Ki-67 can be performed. While the potential effect of this attachment process on the rate of proliferation may not be excluded in the present study, the same procedure applied to all treatment groups. By postnatal day 14, there was a trend for anoxia to decrease proliferation however this trend was not significant in our data. The heart is thought to be fully mature and essentially an adult phenotype of cardiomyocytes by day 14 in rats, therefore the rate of myocyte proliferation is normally very low at this point and anoxia had no significant effect on lowering it further. Similarly, our results from the prenatal hypoxia model showed a decrease in the proliferation of neonatal cardiomyocytes at postnatal day 7. Interestingly, newborn anoxia had no significant effect on the binucleation of cardiomyocytes. Previous work has shown that maternal hypoxia leads to an increase in the amount of binucleate cardiomyocytes in the fetal heart [7], thus indicating a development stage-specific effect.

Furthermore, we investigated two proteins that are closely involved in the regulation of the cell cycle: cyclin D2 and p27. These proteins have previously been studied and found to be differentially expressed in the hypoxia-treated fetal heart [8]. Cyclin D2 is associated with other cell cycle regulators that work to promote cell cycle activity, while p27 is a cyclin-dependent kinase inhibitor and thus inhibits cell cycle activity. Therefore the expression of these two proteins should be inversely related, as our results indicate. Cyclin D2, a cell cycle promoter, is significantly decreased during anoxia treatment, while the cell cycle inhibitor, p27, is upregulated under these conditions. These results are consistent with our finding that anoxia treatment decreases cardiomyocyte proliferation. In addition, we tested the role of ET-1 acting through its ET_A_R on the expression of cyclin D2 and p27. In the presence of PD156707, an ET_A_R antagonist, anoxia had no effect on cyclin D2 expression. However, p27 expression was significantly decreased in the presence of PD156707 compared to control conditions. These findings suggest that ET-1 and the ET_A_R are key mediators in the anoxia-induced effects on cyclin D2 and p27. Ultimately, these results may help to explain the overall decrease in cardiomyocyte proliferation due to anoxia treatment.

Although a gradual decrease in proliferation in the critical window of the heart development is a normal developmental process, hypoxia and anoxia appear to accelerate this progression, particularly during the early development. The endpoint of cardiomyocyte number is a metric to measure the consequence of altering the proliferative capacity. Our results suggest that anoxia reduces cardiomyocyte endowment at postnatal day 14, when the heart is presumed to be fully mature and cardiomyocytes have terminally differentiated. Anoxia reduced proliferation at days 4 and 7, resulting in fewer cardiomyocytes in the differentiated heart seen at day 14. Given that cardiomyocytes are the functional contractile units of the heart, this decreased cardiomyocyte endowment in the heart may have negative impact in cardiac function and become more susceptible to injury later in life. While our results suggest a significant reduction in cardiomyocyte endowment due to anoxia at the critical window of the heart development, future studies using unbiased and random stereology will be needed to provide conclusive evidence of this effect.

Previous studies from our laboratory and others have shown that hypoxia regulates proliferation of cardiomyocytes and vascular muscle [8, 10, 26, 39]. However the downstream regulators of this response have yet to be identified. Our previous work in an *ex vivo* model showed that primary fetal cardiomyocytes exhibited a similar decrease in proliferation when treated with endothelin-1 (ET-1) [26]. It is known that ET-1 expression is induced under hypoxic conditions *via* a HIF-binding site on its promoter [15–20], specifically in cardiomyocytes [21]. ET-1 itself has also been shown to regulate proliferation, having a mitogenic effect on vascular smooth muscle [24, 27, 28]. Moreover our results showed an increase in prepro-ET-1 mRNA in the P4 neonatal heart when exposed to anoxia. Previous work has also shown an increase in prepro-ET-1 mRNA in the fetal heart exposed to maternal hypoxia [26]. These studies taken together implicate a role for ET-1 in mediating the hypoxia- and anoxia-induced decrease in cardiomyocyte proliferation.

A selective ET-receptor antagonist was used to study the role of both basal and anoxia-induced ET-1 in the present study. ET-1 can activate two receptor subtypes: the ET_A_- and ET_B_-receptor. Activation of the ET_A_-receptor leads to vasoconstriction and is primarily found in vascular muscle [40]. In contrast, the ET_B_-receptor can provide a vasodilation effect as well as vasoconstriction depending on the receptor location, in endothelial cells [41] or vascular muscle [41–44], respectively. The ET_B_-receptor also plays a role in the clearance of endothelin from tissues [45]. In cardiomyocytes, the ET_A_-receptor is the predominant subtype [23], and has been implicated in regulating proliferation [24, 27, 28]. Therefore, our study evaluated the effects of PD156707, a selective antagonist for the ET_A_-receptor [46], on cardiomyocyte proliferation. Due to the short half-life of PD156707 of about one hour [47], it was given twice a day just prior to anoxia exposure in the present study. We also evaluated the protein expression of the ET-receptors, both ET_A_R and ET_B_R. Interestingly, the results showed no change in the expression of either receptors due to anoxia treatment, suggesting that a change in receptor density is not contributing to the effects of anoxia or ET-1. The finding that PD156707 ameliorated the anoxia-induced decrease in proliferation of cardiomyocytes at day 4 and 7 implicates the ET_A_-receptor as a key mediator. Furthermore, the addition of PD156707 alone elicited an increase in proliferation at day 4 beyond that of the control. This observation was not seen at day 7 or day 14, suggesting that the regulation of basal ET-1 function in the heart is dependent on the stage of development. At an earlier stage, basal ET-1 levels play a key role in regulating cardiomyocyte proliferation. The effect of basal ET-1 in regulating cardiomyocyte endowment in the developing heart is intriguing. The treatment of newborn rats with ET_A_-receptor antagonist led to an increase in cardiomyocyte number per heart weight at day 7, suggesting that an appropriate level of basal ET-1 is necessary to optimize cardiomyocyte endowment in the heart.

Anoxia treatment had no significant effect on mononucleate and binucleate cell size, however inhibition of ET_A_R by PD156707 caused an increase in cell size at day 7. This may suggest that basal ET-1 plays a role in maintaining cell size and, and if activation of the ET_A_-receptor is blocked, the cell undergoes hypertrophy. The change in binucleate cell size is likely more relevant because the mononucleate cells still have the capacity to divide and are not yet terminally differentiated.

The heart to body weight ratio was unchanged with anoxia treatment for all age groups. However by blocking basal ET-1 with PD156707, the heart to body weight ratio was increased at postnatal day 7. These results suggest that the heart is increasing in size, which agrees with the results of increased cell size, proliferation, and cardiomyocyte number in the presence of PD156707. In the present study, the cardiomyocyte number were counted in freshly isolated myocytes, and the *in vivo* PD156707 treatment increased the cardiomyocyte number by about 65% in day 7 hearts. The heart is made up by cardiac fibroblasts, myocytes, endothelial cells, and vascular smooth muscle cells, with the majority being fibroblasts and myocytes. There are significant differences in cell populations of the heart among various species. In rats, the heart is composed of about 60–70% nonmyocytes and 30–40% cardiac myocytes [48–53]. In neonatal rats, cardiac fibroblasts made up about 64% of the total heart, whereas the myocyte population was 30%, with the nonmyocyte and nonfibroblast cell populations comprising the remainder of the heart [54]. In the same study [54], it was found that neonatal and adult mouse hearts contained around 60% cardiac myocytes. However, in a more recent study, 20–30% cardiac myocytes were demonstrated in the mouse heart [55]. Because of the variability of the myocyte maturity in near-term fetuses and neonates among different species, there is also a dramatic difference in the myocyte volume density of the heart between various species. The near-term heart myocyte volume density, for example, was 53–55% in sheep [56] with highly matured heart at birth, but was much lower in rats of 21–30% [57] and rabbits of 22% [58], the hearts of which were much immature at birth and continued the maturity in the first two weeks of postnatal life. However, a study reported that the myocyte volume density in fetal and neonatal rats was around 80–94% [59]. This was a somewhat surprising finding and it is unlikely that rats would have much higher myocyte volume density than lambs given that matured myocytes in lambs should have larger volume than those of immature myocytes in neonatal rats. In the present study, if the PD156707 treatment induced proportional changes in the nonmyocyte composition of the heart, it would increase the cardiomyocyte composition in the heart to around 50%, albeit the proliferation of nonmyocyte cells in the heart could be differentially regulated. It is important to note that although changes in cardiomyocyte size measured in cells that were attached to plates suggest a physiological difference due to the PD156707 treatment, they are not necessarily representative of what’s happening *in vivo*. The possibility that the PD156707 treatment may cause more rapidly flatten out of myocytes, giving a larger area reading in the 24 hour-period of culture, may not be excluded in the present study. It is likely that the increases of both cardiomyocyte number and cell size may contribute to significantly increased heart weight observed in the PD156707-treated animals. The finding that the heart to body weight ratio is unchanged at day 14 even though anoxia treatment decreases cardiomyocyte endowment implies that the cardiomyocytes may increase their size to compensate for the loss of cells and maintain the size of the heart. However, we were unable to measure cardiomyocyte size from the day 14 hearts due to technical limitations and the difficulty of cardiomyocytes in the attachment to the plate at this stage. Another possibility includes an increase in non-cardiomyocyte cell number and size in the heart after anoxia treatment.

The present study evaluated not only the effects of newborn anoxia treatment on the terminal differentiation of neonatal cardiomyocytes but also the role of basal ET-1 on this process. We identified a mechanism through which neonatal anoxia exposure induces an accelerated loss of cardiomyocyte proliferation *via* the ET_A_-receptor, which subsequently results in reduced cardiomyocyte endowment in the fully differentiated heart. Our study also demonstrated the role that basal ET-1 plays in regulating cardiomyocyte size, proliferation, and number in the developing heart. Given the clinical implications of these findings in understanding the effects of hypoxia on the heart development in preterm infants, further investigation into the mechanisms involved is needed.
